# Synthetic microbial communities: a novel emerging models for dissecting gut microbiota-host interactions in neurodegenerative diseases

**DOI:** 10.3389/fimmu.2026.1822743

**Published:** 2026-05-21

**Authors:** Pengya Feng, Wenqiang Zhang, Yonghong Zhao, Pengju Zhao, Enyao Li

**Affiliations:** 1 Department of Children Rehabilitation, Henan Key Laboratory of Rehabilitation Medicine, The Fifth Affiliated Hospital of Zhengzhou University, Zhengzhou, Henan, China; 2 Henan Key Laboratory for Helicobacter Pylori and Digestive Tract Microecology, The Fifth Affiliated Hospital of Zhengzhou University, Zhengzhou, China

**Keywords:** gut microbiota, immune, mechanisms, neurodegenerative diseases, synthetic microbial communities

## Abstract

The gut-brain axis (GBA) has emerged as a critical regulatory pathway underlying the pathogenesis of neurodegenerative diseases (NDs) such as Alzheimer’s disease and Parkinson’s disease. However, the high complexity and individual variability of native gut microbiotas hinder the precise elucidation of causal relationships between specific microbial taxa, their metabolites, and host neuroinflammatory or neurodegenerative processes. Synthetic microbial communities (SynComs), consisting of defined and reproducible bacterial strains, have recently emerged as powerful experimental models to overcome these limitations. This review summarizes the applications of SynComs in dissecting GBA crosstalk in NDs, highlighting their utility in validating key microbial mediators, deciphering molecular signaling pathways (e.g., microbial metabolite-brain barrier interactions, immune cell activation), and evaluating therapeutic strategies targeting the gut microbiota. By reducing community complexity while retaining core functional traits, SynComs enable controlled *in vitro* and *in vivo* studies that bridge the gap between observational microbiome profiling and mechanistic insights. Furthermore, the customization of SynComs allows for mimicking disease-specific microbial dysbiosis, facilitating the identification of novel therapeutic targets for NDs. Collectively, SynComs represent an innovative and standardized tool to advance our understanding of gut microbiota-host interactions in neurodegeneration and accelerate the development of microbiome-based interventions.

## 1 Introduction

The gut microbiota, a complex and dynamic ecosystem of trillions of microorganisms including bacteria, fungi, archaea, and viruses, residing in the gastrointestinal tract (1). Recently, it is increasingly recognized as a pivotal regulator of human health and disease, exerting far-reaching impacts across multiple physiological systems (2). The gut microbiota and host form a mutualistic, dynamically balanced symbiotic relationship shaped by long-term co-evolution, with interactions spanning nutrient metabolism, immune regulation, neuro-metabolic crosstalk, and mucosal barrier maintenance (3). The host provides the gut microbiota with a viable environment and nutrient sources, while the microbiota acts as a core regulator of host physiological balance and disease development (4). When these gut microbiota-host interactions are in a state of balance, they play a crucial role in preserving host homeostasis and promoting overall physiological fitness (5). Conversely, disruption of this symbiotic balance (dysbiosis)-characterized by reduced microbial diversity, depletion of beneficial bacteria, and overgrowth of pathogens-impairs intestinal barrier function, triggers systemic inflammation, and is linked to the pathogenesis of metabolic disorders, autoimmune diseases, and neuropsychiatric conditions (6).

The gut-brain axis refers to the bidirectional communication network that connects the central nervous system (CNS) with the enteric nervous system (ENS) through neural, humoral, immune, and metabolic pathways (7). Emerging evidence indicates that dysregulation of this axis plays a pivotal role in the pathogenesis of various neurodegenerative diseases, such as Alzheimer’s disease, Parkinson’s disease, and Autism spectrum disorder (8). Gut microbiota, as a core component of the axis, modulates neuroinflammation, neurotransmitter synthesis, and blood-brain barrier permeability, thereby influencing neuronal survival and function in the brain (9). Thus, targeting the gut-brain axis and restoring microbial balance through probiotics, prebiotics, or fecal microbiota transplantation has gained increasing attention as a novel, non-invasive therapeutic approach for managing neurological disorders (10). However, the intricate structural composition and functional complexity of the gut microbiota pose considerable challenges to investigating the mechanisms by which gut-brain axis interventions ameliorate neurological diseases, thereby imposing certain constraints on their clinical application.

Current methodologies for dissecting the gut microbiota-mediated mechanisms of the gut-brain axis fall broadly into two categories. While traditional *in vivo* models-such as germ-free rodents or animals treated with broad-spectrum antibiotics-yield invaluable mechanistic insights, they are inherently limited by a lack of ecological fidelity (11). Similarly, conventional *in vitro* models fall short of recapitulating the spatial, metabolic, and immunological complexity inherent to the intestinal microenvironment (12). Over the past decade, synthetic microbial communities (SynComs) have established themselves as powerful reductionist research tools. By retaining the functionally relevant attributes of indigenous gut microbiota, these precisely defined microbial consortia effectively overcome the experimental bottlenecks that hinder mechanistic investigations into complex natural microbial assemblages (13). As defined microbial consortia composed of axenically cultured isolates, the constituent taxa of SynComs are derived from a broad spectrum of ecological niches spanning soil matrices, plant-associated microenvironments, aquatic systems, and animal host-associated microbiomes (14). In the context of gut microbiota investigations, SynComs have to date been largely assembled using bacterial taxa as core components. These precisely defined microbial consortia offer a robust, experimentally tractable system that enables the accurate delineation of strain-specific functional impacts, interspecies microbial crosstalk, and host adaptive responses under well-standardized, highly reproducible experimental settings (15). Thus, SynComs offer a distinctive equilibrium between ecological validity and mechanistic dissectability, emerging as a powerful tool for exploring host metabolic pathways, immune regulatory mechanisms, and the development of microbiome-directed therapies.

In the present review, we highlight SynComs as a cutting-edge experimental tool for dissecting gut microbiota-host crosstalk underlying neurodegenerative diseases. Specifically, we evaluate key aspects of SynComs, including their construction strategies, inherent methodological strengths and drawbacks, synergistic integration with traditional *in vivo* and *in vitro* models, as well as their translational potential to deepen mechanistic insights and fuel the development of novel therapeutic strategies.

## 2 Microbiota-gut-immune-brain axis and neurodegenerative diseases

**Figure 1:**
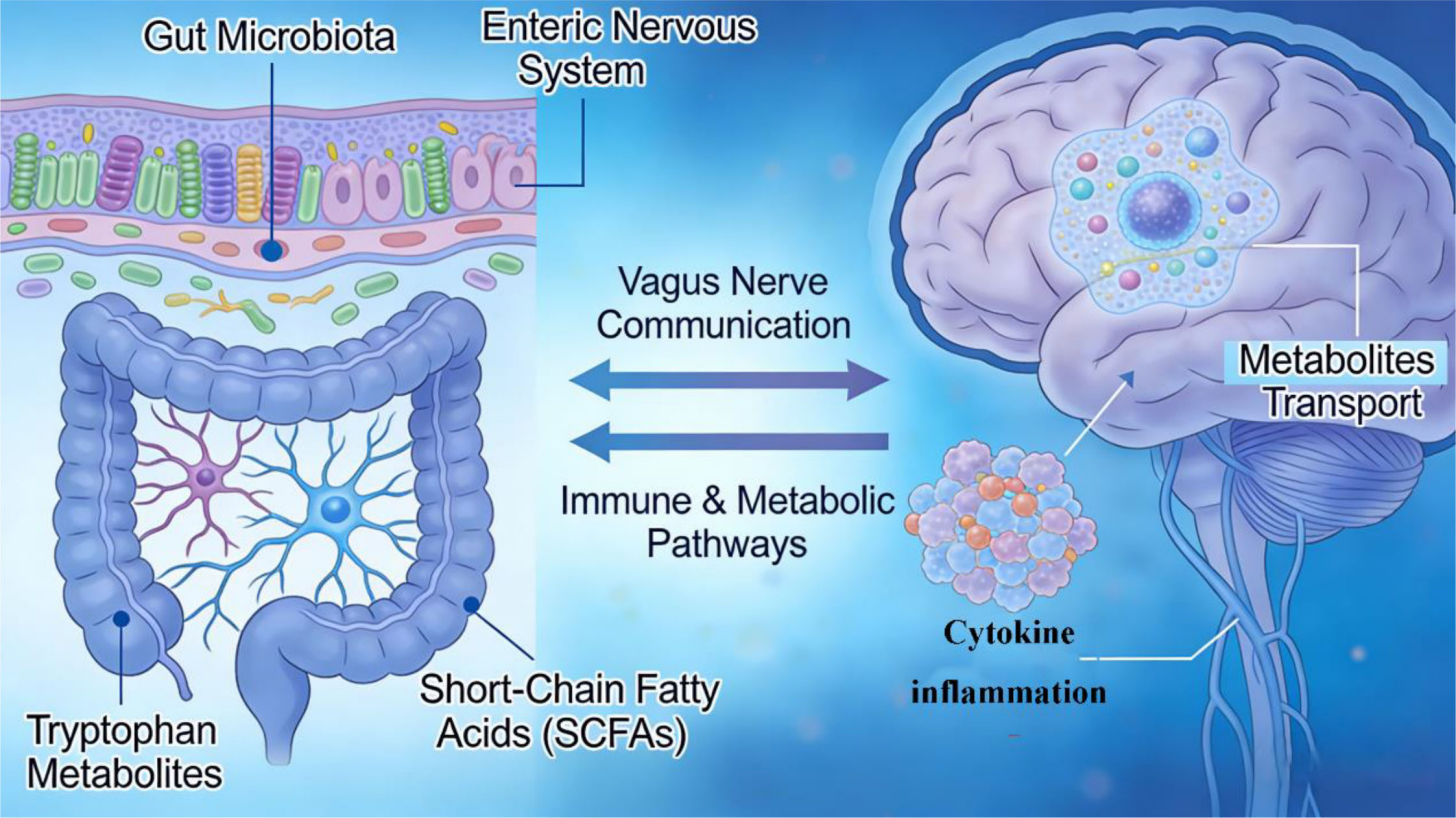
The microbiota-gut-immune-brain axis communication network involves neural, immune, and metabolic pathways. Dysregulation of gut microbiota and neuroinflammation contribute to neurodegenerative disease pathogenesis, including protein misfolding and neurotransmitter imbalance.

Neurodegenerative diseases including Alzheimer’s disease (AD), Parkinson’s disease (PD), and Huntington’s disease (HD) are progressive, irreversible disorders defined by structural and functional deterioration of neuronal populations, culminating in cognitive impairment, motor deficits, and premature mortality (16). Their etiology is heterogeneous and multifactorial, involving inherited genetic variants, aberrant protein aggregation, mitochondrial dysfunction, oxidative damage, and chronic neuroinflammation (17). While traditional research has centered on intrinsic central nervous system (CNS) pathology, contemporary studies have expanded this view to integrate a microbiota−gut−immune−brain axis-a holistic, bidirectional communication network that unites gut microbial ecology, intestinal physiology, systemic immunity, and brain homeostasis (**Figure 1**). This integrated framework supersedes the classical gut-brain axis by explicitly emphasizing immune signaling as a central mechanistic hub, through which gut microbiota regulate microglial reactivity, barrier integrity, and metabolite−mediated neuromodulation, collectively shaping neurodegenerative progression (18).

### 2.1 Immune−mediated neuroinflammation as a central pathogenic hub

Chronic, low−grade neuroinflammation is a unifying pathological hallmark across AD, PD, and HD, driven primarily by dysregulated immune activity within the CNS (19). Unlike acute inflammatory responses that support tissue repair, sustained neuroinflammation promotes synaptic loss, neuronal apoptosis, and enhanced protein misfolding and aggregation. Within the expanded microbiota−gut−immune−brain axis, peripheral immune activation originating in the gut acts as a major upstream trigger for CNS inflammation, establishing a feedforward cycle between intestinal immune dysregulation and neurotoxicity (20).

Microglial Activation and Priming: Microglia, the resident innate immune cells of the brain, are the principal mediators of neuroinflammation and exist in a surveillant, homeostatic state under physiological conditions (21). Perturbations along the microbiota−gut−immune−brain axis induce two key functional states: Classical microglial activation: Direct exposure to pro−inflammatory signals (e.g., microbial products, cytokines) triggers a pro−inflammatory phenotype characterized by secretion of TNF−α, IL−1β, IL−6, reactive oxygen species (ROS), and nitric oxide (NO), which directly damage neurons and exacerbate proteinopathy (22). Microglial priming: Subthreshold inflammatory stimuli from the gut−immune axis render microglia hypersensitive to subsequent CNS insults, such as amyloid−β or α−synuclein aggregates (9). Primed microglia produce exaggerated inflammatory bursts upon secondary challenge, amplifying neurodegeneration without an acute exogenous trigger (23).

Gut microbiota and their metabolites serve as critical regulators of microglial maturation and reactivity. Dysbiotic microbial communities disrupt microglial development in early life and promote a pro−inflammatory, primed phenotype in adulthood, rendering the brain more vulnerable to age−related neurodegeneration.

### 2.2 Barrier permeability: intestinal barrier dysfunction and blood-brain barrier leakage

A functionally intact intestinal barrier and blood-brain barrier (BBB) are essential for isolating systemic immune and microbial signals from the CNS. In the microbiota−gut−immune−brain axis, microbiota−driven barrier disruption creates a pathophysiological conduit for peripheral inflammation to invade the CNS (24). Intestinal Barrier Permeability (“Leaky Gut”): The intestinal epithelium forms a selective barrier maintained by tight junction proteins (e.g., occludin, claudins, zonulin). Gut dysbiosis reduces the abundance of beneficial commensals that support barrier integrity while enriching pro−inflammatory pathobionts. This imbalance increases intestinal permeability, enabling the translocation of microbial components including lipopolysaccharide (LPS), peptidoglycans, and pathogenic antigens into the systemic circulation (25). The resulting low−grade endotoxemia activates systemic immune cells and promotes the release of pro−inflammatory cytokines, initiating a cascade that targets the brain. Blood-Brain Barrier Permeability: The BBB, composed of endothelial cells, pericytes, and astrocytic endfeet, normally restricts the entry of circulating cytokines, immune cells, and microbial metabolites (26). Chronic systemic inflammation induced by leaky gut upregulates adhesion molecules on brain endothelial cells, weakens tight junctions, and increases BBB permeability. This allows pro−inflammatory mediators and even peripherally activated immune cells to infiltrate the CNS parenchyma, directly triggering microglial activation and neuroinflammation. Barrier dysfunction at both the gut and BBB thus acts as a mechanistic bridge in the microbiota−gut−immune−brain axis (27).

### 2.3 Microbial metabolite signaling: immune and neural modulators in the axis

Gut microbiota function as a metabolic organ that converts dietary substrates into various small-molecule metabolites. Acting as key signaling molecules, these metabolites modulate immune cell activity, intestinal barrier integrity and neuronal health, connecting microbial ecology to CNS physiology (28). Major immune-active metabolites include short-chain fatty acids (SCFAs), bile acid derivatives and tryptophan metabolites.

SCFAs (mainly acetate, propionate, butyrate) are produced by microbial fermentation of dietary fiber and play multiple regulatory roles in the microbiota-gut-immune-brain axis. They supply energy for colonocytes, upregulate tight junction proteins and reduce intestinal permeability (29). SCFAs also inhibit histone deacetylases (HDACs) and activate G-protein-coupled receptors (GPR41, GPR43, GPR109A), suppressing pro-inflammatory activation of macrophages and microglia while promoting anti-inflammatory Treg differentiation (30). Moreover, SCFAs cross the blood-brain barrier, enhance BDNF expression, alleviate oxidative stress and limit pathological protein aggregation. SCFA deficiency caused by dysbiosis impairs these protective effects, leading to barrier dysfunction, systemic inflammation and microglial priming (31). Gut microbiota mediate the conversion of primary bile acids into secondary bile acids (e.g., deoxycholic acid, lithocholic acid). These bile acids signal through host receptors such as FXR and TGR5, regulating intestinal inflammation, innate immune responses and microglial function. Microbiota-related bile acid metabolic disorders disrupt homeostasis, intensify inflammation and induce pro-inflammatory microglial phenotypes, contributing to neurodegeneration (32, 33). Gut microbiota metabolize dietary tryptophan into kynurenine, serotonin precursors and aryl hydrocarbon receptor (AhR) ligands. AhR activation in intestinal immune cells and microglia exerts anti-inflammatory effects by inhibiting pro-inflammatory cytokines and preserving barrier function. Dysbiosis reduces the generation of beneficial AhR ligands and diverts metabolism toward neurotoxic kynurenine pathway intermediates. This imbalance aggravates neuroinflammation, disturbs neurotransmitter homeostasis and increases oxidative stress in the CNS (34). This imbalance exacerbates neuroinflammation, impairs neurotransmitter homeostasis, and promotes oxidative stress in the CNS.

## 3 SynComs design and applied for mechanism investigation

### 3.1 SynComs design and construction

Microbial communities, as complex assemblages of interacting microorganisms, play pivotal roles in diverse ecological niches, from the human gut to soil and aquatic ecosystems. However, the inherent complexity of natural microbial communities-characterized by countless unknown species and intricate interspecies interactions-poses significant challenges to dissecting their functional mechanisms (35). In response to this limitation, SynComs have emerged as powerful reductionist tools. Defined as deliberately assembled consortia of cultured, genetically defined microorganisms, SynComs retain the key functional attributes of indigenous microbiota, while simultaneously enabling rigorous and targeted experimental manipulation, offering unprecedented opportunities to unravel microbial interactions, elucidate functional mechanisms, and develop tailored microbial-based applications (36).

**Figure 2:**
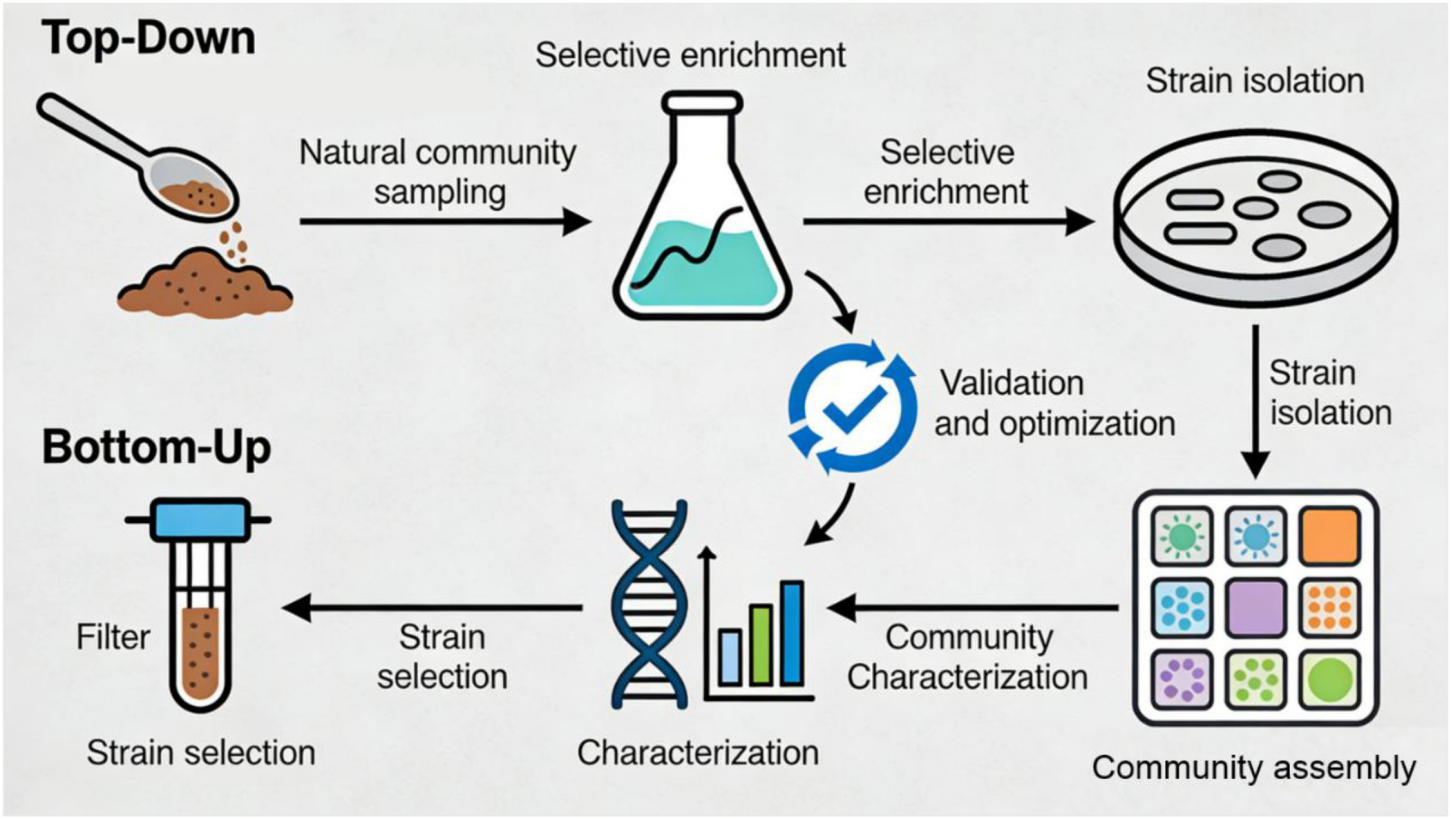
Schematic overview of synthetic microbial community construction approaches. Top-downmethod involves natural community enrichment and strain isolation; Bottom-up strategy focuses on *de novo* assembly of characterized strains.

The design of SynComs is a systematic process guided by clear objectives and ecological principles, laying the foundation for functional stability and reproducibility (37). The primary step in SynCom design is defining the target function, which dictates the selection of constituent microorganisms. Common objectives include simulating native microbial functions, producing valuable metabolites, or dissecting specific interspecies interactions (38). A foundational principle governing strain selection is metabolic complementarity, which ensures that members of the consortium fulfill non-redundant roles and support one another through cross-feeding. This strategy leverages the metabolic specialization of individual strains, where byproducts or essential nutrients (e.g., amino acids, vitamins, or carbon sources) produced by one strain serve as growth substrates for another. For example, in a cellulose-degrading SynCom, a strain capable of breaking down complex polysaccharides can supply simple sugars to a secondary strain that converts these sugars into valuable chemicals, creating an obligate mutualism. Such metabolic interdependencies prevent the competitive exclusion of slower-growing strains and drive the emergence of community-level functions unattainable by monocultures. Furthermore, genomic-scale metabolic models are increasingly employed to predict pairwise metabolic exchanges, enabling the rational selection of strains with maximal complementary pathways to optimize resource utilization and minimize waste accumulation. Another core principle in SynCom design is optimizing community composition and structure. The number of constituent strains, their relative abundances, and spatial arrangement are critical factors influencing community stability and function (39). Concomitant with metabolic complementarity, ensuring the ecological stability of simplified microbial consortia is paramount for long-term functionality. Stability is often achieved by selecting strains with compatible growth kinetics and by fostering a network of positive interactions (mutualism, commensalism) that outweigh competitive pressures. A key ecological strategy is the inclusion of keystone species-strains that, despite potentially low abundance, exert a disproportionate influence on community structure by providing essential public goods (e.g., detoxification of inhibitors, nutrient cycling) or by modulating interspecies signaling. Additionally, stability can be enhanced by incorporating functional redundancy, where multiple strains contribute to the same critical function, buffering the community against the loss of individual members. Mathematical models further suggest that stable consortia adhere to rules of ‘flux conservation’ (balanced production and consumption of resources) and ‘differential resource limitation’ (each strain limited by a unique factor), preventing any single strain from dominating. Reductionist approaches often start with simple two-species or three-species consortia to dissect pairwise or trilinear interactions, then gradually scale up to more complex communities (40). At present, the construction of SynComs involves a series of standardized experimental steps, and two predominant approaches have been established: top-down and bottom-up (41) (**Figure 2**). The top-down approach starts with complex natural microbial communities, followed by selective enrichment, isolation, and screening to obtain a simplified consortium that retains the target functional trait. This method leverages the inherent ecological adaptability of native microbial assemblages, ensuring high functional relevance (42). A critical consideration in both top-down and bottom-up strategies is the influence of host diet and environmental context on strain performance. Strains must be selected not only for their intrinsic metabolic capabilities but also for their ability to thrive and cooperate under the specific abiotic and biotic conditions of the target environment. For host-associated SynComs (e.g., gut or plant rhizosphere), strains must be adapted to the host’s diet, pH, oxygen tension, and immune factors. For instance, gut SynComs designed for a high-fiber diet require strains with specialized carbohydrate-active enzymes (CAZymes) to degrade complex plant polysaccharides, while those for a high-fat diet would prioritize strains capable of metabolizing bile acids and saturated fats. Similarly, environmental SynComs for soil or water remediation must be screened for tolerance to relevant stressors (e.g., heavy metals, salinity, or pH extremes) present in the contaminated site. Selecting strains indigenous to the target environment often improves colonization success and functional stability, as they are pre-adapted to the local ecological context. In contrast, the bottom-up approach-also known as the *de novo* assembly method-relies on the deliberate selection and combination of individually characterized pure strains. This strategy offers precise control over community composition, facilitating the dissection of specific interspecies interactions. The general construction workflow, regardless of the approach, proceeds from strain preparation to community assembly and validation (43). For the top-down approach, the first step involves the collection of natural microbial samples, followed by selective enrichment culture using media tailored to the target function. Subsequent isolation and purification of strains are conducted via repeated streaking, and strains are then screened to identify those contributing to the target function (44). For the bottom-up approach, the initial step is the direct isolation and purification of candidate strains from natural environments or existing strain collections using selective media. Regardless of the approach, each strain is subsequently characterized using phenotypic and genotypic methods to confirm identity and functional traits. This characterization step is crucial to ensure that each strain contributes to the target function and does not exhibit harmful or antagonistic properties (45).

Following strain preparation, the next step is community assembly, which also varies slightly between the two approaches. For the bottom-up approach, the most common assembly method is the “mixing method,” where pure cultures of selected strains are grown to exponential phase, quantified using techniques such as optical density (OD) measurement or flow cytometry, and then mixed in predefined ratios in a suitable growth medium. This method is simple and reproducible, making it widely used in basic research (46). For the top-down approach, assembly typically involves combining the screened and purified strains from the enriched natural community, often adjusting their relative abundances based on their functional contributions observed during enrichment. For more complex applications requiring spatial structure-applicable to both approaches-microfluidic devices and 3D bioprinting techniques are employed to control the spatial arrangement of strains, mimicking the spatial heterogeneity of natural microbial communities (47). Additionally, genetic engineering tools can be integrated into SynCom construction (more commonly in the bottom-up approach due to the well-characterized genetic background of pure strains) to enhance functional performance (48).

Given the inherent complexity of SynComs-encompassing intricate metabolic interactions, dynamic community dynamics, and context-dependent functionality-computational and systems biology approaches have become indispensable tools to strengthen the rational design, prediction, and optimization of synthetic consortia (49). These tools complement experimental methods by providing quantitative insights into community behavior, reducing trial-and-error in strain selection and assembly, and enhancing the reproducibility of SynCom functionality.

Genome-scale metabolic models (GEMs) and flux balance analysis (FBA) are foundational computational tools for predicting metabolic interactions within SynComs. GEMs integrate genomic data to map the complete set of metabolic reactions, enzymes, and metabolites of individual strains, while FBA quantifies the flow of metabolites through these pathways under steady-state conditions (50). When applied to multi-strain consortia, GEMs and FBA enable the prediction of cross-feeding interactions (e.g., metabolite exchange between strains), identify metabolic bottlenecks, and optimize strain combinations to maximize target functions (e.g., metabolite production or nutrient degradation) (51). For instance, FBA can predict how the metabolic flux of one strain influences the growth and function of another, guiding the selection of strains with complementary metabolic pathways as discussed earlier. Beyond metabolic modeling, multi-species and community assembly models provide critical insights into SynCom stability and interspecies dynamics. These models, often based on ecological principles (e.g., competition, mutualism, and niche partitioning), simulate how strain abundances change over time, predict the likelihood of competitive exclusion or coexistence, and identify factors that maintain community stability (52). Such models help refine strain selection by highlighting combinations of strains with compatible growth kinetics and positive interaction networks, aligning with the ecological stability principles of SynCom design.

Machine learning (ML) approaches further enhance SynCom design by leveraging large datasets (e.g., genomic, transcriptomic, and metabolomic data) to predict strain compatibility, optimize community composition, and even identify novel strain combinations with desired functions (53). ML algorithms can learn patterns from existing SynCom data to classify strains based on their functional contributions, predict community-level phenotypes from individual strain traits, and reduce the computational burden of simulating complex multi-strain interactions (54). Additionally, network analysis of metabolite flow-mapping the direction and magnitude of metabolite exchange between strains-provides a visual and quantitative framework to understand interspecies dependencies, identify keystone metabolites, and refine strain selection to strengthen mutualistic interactions (55).

Validation and optimization are essential final steps in SynCom construction. Assembled SynComs are cultured under controlled conditions, and their stability and functional performance are monitored (48). High-throughput sequencing is used to confirm that the community composition matches the designed ratio and remains stable during cultivation (56). Functional validation is performed using targeted assays-for example, measuring the production of a specific metabolite or evaluating the ability of a gut-derived SynCom to modulate host immune responses (57). If the SynCom fails to meet the desired criteria, optimization is conducted by adjusting strain ratios, modifying growth conditions, or adding/removing strains based on interaction data. As the design and construction methods continue to refine, SynComs will undoubtedly become increasingly powerful tools for understanding and harnessing microbial communities.

### 3.2 Advantages of SynCom in studying microbial interactions

Studies exploring gut microbiota-host crosstalk have long depended on diverse traditional methods, each yielding unique mechanistic insights while also harboring unavoidable limitations (58).

Traditional microbiome research methods, such as 16S rRNA gene sequencing, metagenomics, and culturing-dependent isolation, have contributed significantly to our initial understanding of microbiomes. 16S rRNA gene sequencing enables the profiling of microbial community composition (59), while metagenomics provides insights into the potential functional genes encoded by the community (60). Culturing-dependent methods allow for the isolation and characterization of individual microbial strains (61). Nevertheless, these methods are insufficient for studying microbial interactions. For instance, 16S rRNA gene sequencing and metagenomics can only provide correlative evidence of microbial co-occurrence, failing to establish causal relationships between specific taxa and their interactions (62). Culturing-dependent methods, on the other hand, often isolate strains in monoculture, which cannot replicate the complex interspecies interactions that occur in natural communities (63). Additionally, traditional methods are limited by the “great plate count anomaly”-the fact that most microbial strains in natural environments cannot be cultured under laboratory conditions-leading to an incomplete representation of the actual microbial community (64). In contrast, SynCom overcome these limitations by offering high controllability over community composition, which is crucial for dissecting specific microbial interactions (65). SynCom are constructed by assembling a set of known, cultivable microbial strains with defined genotypes and phenotypes. Researchers can precisely manipulate the number of taxa, the relative abundance of each strain, and even the genetic background of individual strains (e.g., through gene knockout or overexpression). This controllability allows for targeted studies of pairwise or multi-species interactions (48). For example, by constructing synthetic communities containing different combinations of two or three strains, researchers can directly observe the positive (e.g., mutualism, commensalism) or negative (e.g., competition, antagonism) interactions between specific taxa, and quantify the impact of these interactions on community stability and function (66). Such targeted manipulation is nearly impossible with traditional methods, which rely on natural communities with undefined compositions.

**Figure 3:**
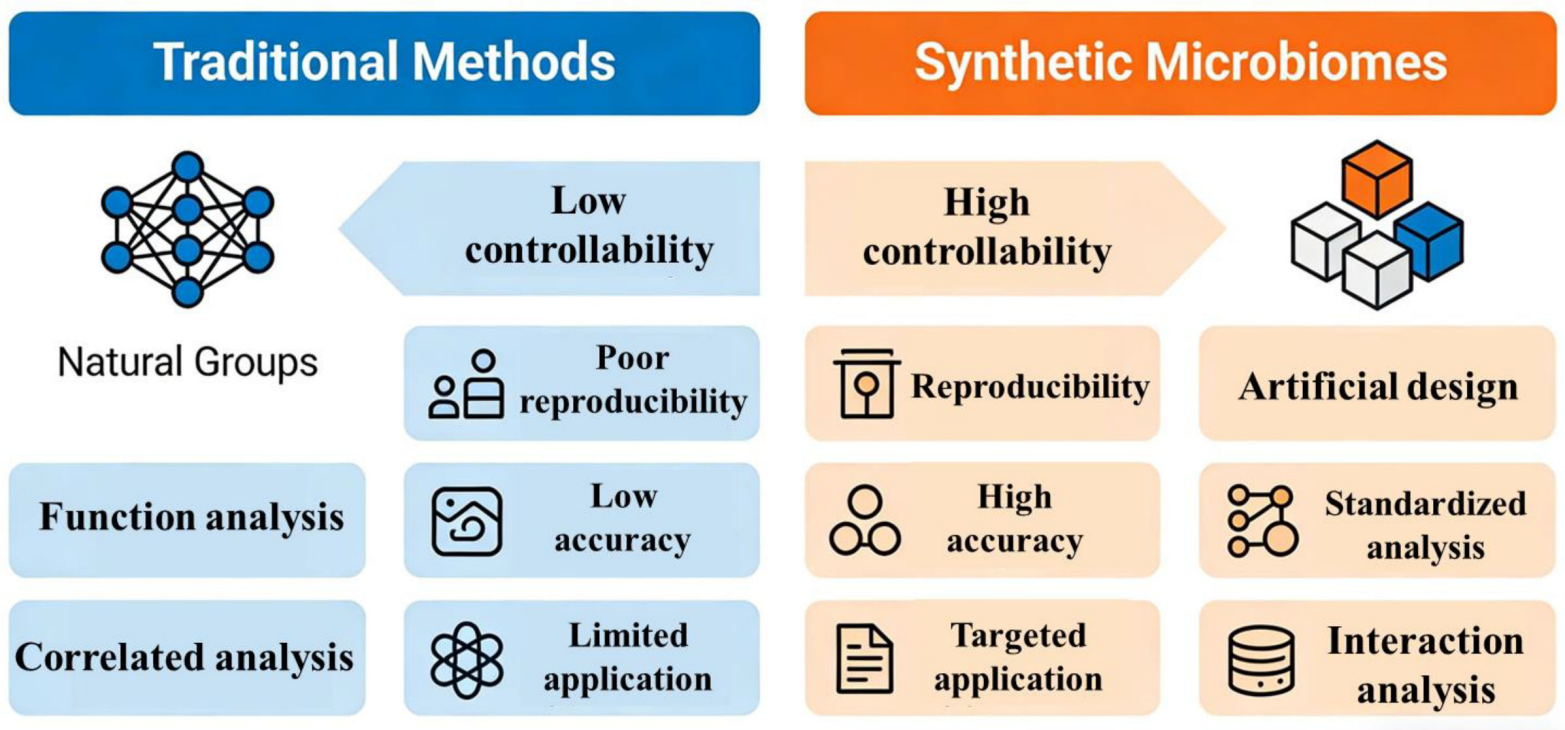
The comparison of traditional methods with synthetic microbial communities in microbial international analysis, highlighting the key differences in controllability, reproducibility and application.

Another key advantage of SynCom is enhanced reproducibility, a critical factor in scientific research. Natural microbial communities are highly variable, even across seemingly similar environments (67). For example, two soil samples from the same field or two gut microbiota samples from healthy individuals can differ significantly in composition, making it difficult to replicate experiments and validate results (68). SynCom, however, are standardized and can be precisely reconstructed in different laboratories, ensuring that experiments are reproducible. This reproducibility allows researchers to systematically investigate the effects of specific variables (e.g., environmental conditions, strain genotypes) on microbial interactions, leading to more reliable conclusions (38). In contrast, traditional studies using natural communities often suffer from low reproducibility due to the inherent variability of the samples, hindering the advancement of knowledge about microbial interaction mechanisms (69). SynCom also excel in unraveling the molecular mechanisms of microbial interactions, which is a major goal of microbiome research. Traditional methods can identify potential interaction-related genes through metagenomic sequencing, but they cannot directly link these genes to specific interspecies interactions (70). SynCom, combined with genetic engineering tools, enable researchers to dissect the molecular basis of interactions. For example, by constructing mutant strains of a specific taxon in a synthetic community, researchers can determine which genes are involved in the production of signaling molecules, antibiotics, or nutrients that mediate interactions with other strains (71). Additionally, omics technologies (e.g., transcriptomics, metabolomics) can be applied to SynCom to profile the gene expression and metabolite production of each strain during interaction, providing a comprehensive understanding of the molecular pathways underlying microbial interactions (72). This level of mechanistic insight is difficult to achieve with traditional methods, which often provide only superficial information about community composition and function (73). Furthermore, SynCom offer the ability to simulate and predict natural microbial interactions more accurately. While natural communities are complex, synthetic microbial communitiess can be constructed as simplified models of natural systems (37). By gradually increasing the complexity of SynCom (e.g., adding more strains that are present in natural environments), researchers can simulate the gradual formation and evolution of microbial interactions in natural communities. This incremental approach allows for the identification of key taxa and interactions that drive community function, which can then be validated in natural environments (74). In contrast, traditional methods often study natural communities as a whole, making it difficult to distinguish between key and peripheral interactions (75). SynCom thus serve as a bridge between simplified laboratory models and complex natural systems, facilitating the translation of laboratory findings to real-world applications. Finally, SynCom have greater potential for applications based on microbial interactions (76). Once the mechanisms of beneficial microbial interactions are elucidated using synthetic microbial communitiess, researchers can construct functional synthetic communities for practical use. For example, in agriculture, synthetic microbial communitiess consisting of plant-growth-promoting rhizobacteria (PGPR) with synergistic interactions can be developed to enhance crop yield and resistance to pests and diseases (77). In medicine, synthetic gut microbiotas can be designed to restore the balance of the gut microbiota and treat diseases such as inflammatory bowel disease (IBD) by leveraging beneficial interspecies interactions (78). Traditional methods, which focus on describing natural communities, are less capable of providing the precise knowledge needed for such targeted applications (79). In conclusion, SynCom, with their high controllability, enhanced reproducibility, ability to unravel molecular mechanisms, capacity to simulate natural interactions, and potential for practical applications, offer significant advantages in studying microbial interactions when compared to traditional methods (**Figure 3**). As synthetic biology and microbial ecology continue to advance, SynCom will undoubtedly play an increasingly important role in deepening our understanding of microbial interactions and driving the development of microbiome-based technologies.

SynCom offer a powerful, reductionist framework to dissect and validate the causal role of the gut microbiota in modulating behavioral outcomes related to social function, stress, and anxiety (80). By enabling precise, controlled reconstitution of germ-free (GF) mice with defined sets of microbes-rather than relying on complex, variable fecal transplants-SynComs address a key limitation in microbiome-brain research: the inability to disentangle correlative from causal relationships. Studies demonstrate that GF mice exhibit robust social deficits, heightened anxiety-like behaviors, and dysregulated stress responses, such as elevated corticosterone levels and altered activity in hypothalamic-pituitary-adrenal (HPA) axis-related brain regions (81). Reconstitution with well-characterized SynComs can reverse these phenotypes, providing direct evidence for microbial causation. For instance, SynComs engineered to produce SCFAs or neurotransmitters like gamma-aminobutyric acid (GABA) have been shown to normalize anxiety-like behaviors in GF mice, while SynComs containing specific probiotic strains (e.g., *Lactobacillus reuteri*) restore social interaction and modulate neuroplasticity in reward-related brain regions via vagus nerve signaling (82). Moreover, SynComs facilitate the dissection of mechanistic pathways by allowing the addition or subtraction of individual strains, enabling researchers to pinpoint how specific microbes influence neuroinflammation, immune function, and metabolic signaling along the microbiota-gut-brain axis (83). This precision makes SynComs invaluable for modeling neurodevelopmental disorders like autism spectrum disorder (ASD), where microbiota dysbiosis is linked to social impairments; SynComs can be tailored to test whether restoring beneficial microbial functions (e.g., oxytocin modulation or metabolite production) rescues social deficits in animal models (84). Beyond preclinical modeling, SynComs hold translational potential for developing targeted live biotherapeutics to mitigate stress, anxiety, and social behavior abnormalities by precisely engineering microbial communities to regulate host neurobiology.

### 3.3 Applications of SynComs for studying microbiome–host interactions in Neurodegenerative diseases

In recent years, SynCom defined as artificially constructed assemblages of known microbial strains with defined compositions, have emerged as powerful tools to unraveling microbiome-host interactions in neurodegenerative diseases and their implications for therapeutic development (70).

One of the primary applications of SynComs is to identify keystone microbial taxa and their functional metabolites (e.g., SCFAs, bile acids, tryptophan-derived metabolites, and microbial neurotransmitter precursors) that modulate neurodegenerative phenotypes, as these metabolites are implicated in regulating host neurophysiology and neurodegenerative processes. Unlike complex native microbiomes, SynComs enable precise manipulation of microbial composition, helping researchers establish causal relationships between specific microbes, their metabolites, and host responses (83). For example, in AD research, a study constructed a SynCom of 10 bacterial strains (including Bacteroides fragilis, Enterococcus faecalis, and Lactobacillus plantarum) isolated from AD patients and healthy controls. When transplanted into germ-free (GF) mice, the AD-derived SynCom induced cognitive impairment, increased hippocampal Aβ deposition, and upregulated brain pro-inflammatory cytokines (TNF-α, IL-6) compared to the healthy-derived SynCom-effects partially mediated by altered levels of these key metabolites. Mechanistic studies showed that Bacteroides fragilis in the AD SynCom produced high levels of TMAO (a metabolite promoting neuroinflammation and Aβ aggregation (85) and dysregulated other key metabolites. This study demonstrated SynComs’ ability to pinpoint pathogenic microbial taxa and their metabolites in AD progression, establishing a causal link hard to achieve with native microbiomes.

In PD research, SynComs have explored the gut microbiota’s role in α-synuclein aggregation (a PD hallmark (80), with evidence that the aforementioned microbial metabolites contribute to this process. PD patients exhibit gut microbiota alterations (increased Enterobacteriaceae, decreased Blautia and Faecalibacterium) linked to disrupted metabolite profiles. A PD-SynCom (12 strains representative of PD gut microbiota) and a healthy control SynCom were developed; transplanting PD-SynCom into GF mice expressing human α-synuclein enhanced α-synuclein phosphorylation/aggregation in the gut and brain, along with PD-like motor deficits, potentially driven by metabolite imbalances. In contrast, the healthy SynCom suppressed pathology and improved motor function via beneficial metabolites. Notably, Enterococcus faecalis was identified as a key pathogenic contributor in PD-SynCom, as its individual transplantation recapitulated PD-like phenotypes (including metabolite dysregulation), while its depletion abrogated these effects. This work highlights SynComs’ utility in dissecting microbial taxa’s hierarchical roles in neurodegeneration, particularly via modulating functional metabolites.

SynComs also facilitate the exploration of microbial-host immune interactions that drive neuroinflammation, a common feature of neurodegenerative diseases. The gut microbiota modulates the systemic immune system, and dysregulated immune responses can cross the blood-brain barrier (BBB) to induce neuroinflammation (80). For example, in ALS, a study constructed a SynCom based on the gut microbiota of ALS patients, which was enriched in Desulfovibrio and Clostridium species. When transplanted into a mouse model of ALS (SOD1 mice), the ALS-SynCom accelerated disease progression, increased microglial activation in the spinal cord, and reduced the survival rate. Mechanistically, the ALS-SynCom enhanced the production of lipopolysaccharide (LPS) and short-chain fatty acids (SCFAs) imbalances, which activated the TLR4/NF-κB signaling pathway in gut epithelial cells, leading to systemic inflammation and subsequent neuroinflammation. In contrast, a SynCom composed of anti-inflammatory strains (e.g., *Bifidobacterium longum, Faecalibacterium prausnitzii*) attenuated neuroinflammation and delayed ALS progression in SOD1 mice (86). This study highlights how SynComs can be used to dissect the immunomodulatory roles of the microbiome in neurodegeneration and identify protective microbial consortia.

Furthermore, SynComs hold great promise for the development of targeted microbiome-based therapies for neurodegenerative diseases. By identifying protective microbial taxa or consortia, researchers can design SynComs with specific functional properties to restore microbiome homeostasis and alleviate disease phenotypes (87). For example, based on the findings that healthy gut microbiotas suppress AD-related pathology, a recent study constructed a “protective SynCom” consisting of *Lactobacillus rhamnosus*, *Bifidobacterium breve*, and *Akkermansia muciniphila*. Administration of this SynCom to AD mouse models reduced Aβ deposition, improved cognitive function, and downregulated neuroinflammatory markers. Similarly, in PD, a SynCom composed of *Blautia producta* and *Faecalibacterium prausnitzii* was shown to reduce α-synuclein aggregation and improve motor function by restoring SCFA production and suppressing gut inflammation (2). These studies demonstrate the translational potential of SynComs as novel therapeutic agents for neurodegenerative diseases.

## 4 Challenges and future perspectives

Despite their significant advantages, the application of SynComs in neurodegenerative disease research also faces challenges, while also holding great promise for future advancements.

One of the primary challenges lies in simulating the complexity and functional redundancy of natural gut microbiotas (88). Natural gut microbiotas consist of hundreds of microbial species with intricate synergistic and antagonistic interactions, whereas current SynComs are often simplified to dozens of strains. This simplification may fail to recapitulate the full spectrum of microbial metabolites and signaling pathways that modulate host neurophysiology. This gap between simplified experimental models and the clinical reality of human gut ecosystems represents a major translational bottleneck, as findings from overly reductionist SynCom systems often lack predictive value for human therapeutic outcomes. Additionally, the dynamic adaptability of SynComs *in vivo* remains inadequate. The gut microenvironment of ND patients is characterized by altered pH, mucus secretion, and immune responses, which can disrupt the stability of SynComs and lead to inconsistent colonization efficiency, affecting the reproducibility of research results. Poor colonization and stability in the human gut further impede the bench-to-bedside translation of SynComs, as inconsistent engraftment leads to variable pharmacodynamic effects and undermines clinical trial reliability. Another major hurdle is the incomplete understanding of the mechanisms linking SynComs to neurodegeneration (89). The gut-brain axis (GBA) serves as the key pathway for microbiome-host crosstalk, involving microbial metabolites (e.g., short-chain fatty acids, lipopolysaccharides), immune mediators, and neural signaling. However, how specific SynCom components regulate these GBA pathways to influence neuroinflammation, protein misfolding, and neuronal loss in NDs remains unclear (90). This mechanistic ambiguity hinders the rational design of SynCom therapeutics and complicates the establishment of clinically relevant biomarkers to monitor treatment efficacy in translational studies. Moreover, individual differences in host genetics, diet, and lifestyle further complicate the translation of SynCom-based research findings to clinical applications (91), as a SynCom that is effective in animal models or a subset of patients may not be universally applicable. Such inter-individual variability necessitates a shift toward personalized microbiome-based strategies, a cornerstone of precision medicine that requires robust clinical validation across diverse patient populations.

Despite these challenges, the future of SynComs in ND research is promising, with several notable development trends. First, the precision construction of SynComs will be enhanced by integrating multi-omics data (metagenomics, transcriptomics, and metabolomics). By analyzing the gut microbiota profiles of ND patients and healthy individuals, researchers can identify key functional microbes and metabolites, then design SynComs that mimic the functional characteristics of disease-associated or protective microbiomes (37). This precision approach will improve the relevance of SynCom models to clinical scenarios. Multi-omics-driven SynCom design is critical for translational success, as it aligns synthetic microbial functions with the specific pathophysiological signatures observed in patient cohorts, thereby increasing the likelihood of therapeutic efficacy in humans.

Second, the combination of SynComs with advanced *in vitro* and *in vivo* models will deepen the mechanistic understanding of GBA crosstalk (48). For example, microfluidic-based gut-brain organoid systems can simulate the physical and chemical microenvironments of the gut and brain, enabling real-time observation of SynCom-host interactions at the cellular and molecular levels. In addition, genetically modified SynComs, engineered to express fluorescent reporters or specific metabolites, will provide powerful tools to trace microbial colonization, metabolite production, and their effects on neuronal function. These advanced models serve as pivotal translational bridges, allowing for the preclinical validation of SynCom safety, colonization dynamics, and mechanism of action before costly human trials.

Finally, SynComs hold great potential for the development of novel therapeutic strategies for NDs (38). Probiotic SynComs designed to restore gut microbial balance, reduce neuroinflammation, or enhance neuroprotective metabolite production may serve as targeted therapeutics. Furthermore, personalized SynCom therapies, tailored to individual patient microbiome profiles and genetic backgrounds, are expected to improve treatment efficacy and reduce adverse reactions. As live biotherapeutic products (LBPs), SynComs represent a new class of investigational drugs requiring rigorous clinical development pathways, including Phase I safety studies, Phase II efficacy trials, and Phase III confirmatory trials to meet regulatory standards for clinical approval. Clinical trials evaluating the safety and efficacy of SynCom-based therapies in ND patients will be a key focus of future research. Successful translation will also require the establishment of standardized protocols for SynCom manufacturing, quality control, and long-term storage to ensure product consistency and scalability for clinical use.

## 5 Conclusion

In summary, SynComs emerge as a transformative tool for dissecting gut microbiota-host crosstalk in neurodegenerative diseases. Unlike complex native microbiomes, SynComs enable precise manipulation of taxa composition, abundance, and functional traits, facilitating the identification of keystone microbes and their metabolites that modulate neuroinflammation, blood-brain barrier integrity, and neurotransmitter homeostasis. Recent studies demonstrate that SynCom-based models can recapitulate key ND-associated phenotypes, from gut dysbiosis to cognitive decline, while overcoming the limitations of gnotobiotic animal models in translational relevance. Further integration of multi-omics technologies and in silico modeling with SynComs will advance our understanding of causal relationships between specific microbial consortia and ND pathogenesis. Ultimately, rational design of SynComs holds great promise for developing targeted microbiome-based diagnostics and therapeutics to mitigate the burden of neurodegenerative disorders.

## References

[1] Minagar A Jabbour R . The human gut microbiota: a dynamic biologic factory. Adv Biochem Eng/Biotechnol. (2025) 189:91–106. doi: 10.1007/10_2023_243. PMID: 38337077

[2] Zhang Y Wang H Sang Y Liu M Wang Q Yang H . Gut microbiota in health and disease: advances and future prospects. MedComm (2020). (2024) 5:e70012. doi: 10.1002/mco2.70012. PMID: 39568773 PMC11577303

[3] Padhi P Worth C Zenitsky G Jin H Sambamurti K Anantharam V . Mechanistic insights into gut microbiome dysbiosis-mediated neuroimmune dysregulation and protein misfolding and clearance in the pathogenesis of chronic neurodegenerative disorders. Front Neurosci. (2022) 16:836605. doi: 10.3389/fnins.2022.836605. PMID: 35281490 PMC8914070

[4] Alswat AS . The influence of the gut microbiota on host health: a focus on the gut-lung axis and therapeutic approaches. Life (Basel). (2024) 14(10):1279. doi: 10.3390/life14101279. PMID: 39459579 PMC11509314

[5] Malard F Dore J Gaugler B Mohty M . Introduction to host microbiome symbiosis in health and disease. Mucosal Immunol. (2021) 14:547–54. doi: 10.1038/s41385-020-00365-4. PMID: 33299088 PMC7724625

[6] Shen Y Fan N Ma SX Cheng X Yang X Wang G . Gut microbiota dysbiosis: pathogenesis, diseases, prevention, and therapy. MedComm (2020). (2025) 6:e70168. doi: 10.1002/mco2.70168. PMID: 40255918 PMC12006732

[7] Lu S Zhao Q Guan Y Sun Z Li W Guo S . The communication mechanism of the gut-brain axis and its effect on central nervous system diseases: a systematic review. Biomedicine Pharmacotherapy. (2024) 178:117207. doi: 10.1016/j.biopha.2024.117207. PMID: 39067168

[8] Chen J Xu Y Cao P . The role of the gut-brain axis in diseases. Physiology. (2025) 41(2):109–21. doi: 10.1152/physiol.00016.2025 40828621

[9] Park JC Chang L Kwon H-K Im S-H . Beyond the gut: decoding the gut–immune–brain axis in health and disease. Cell Mol Immunol. (2025) 22:1287–312. doi: 10.1038/s41423-025-01333-3 PMC12575876 40804450

[10] Origüela V Lopez-Zaplana A . Gut microbiota: an immersion in dysbiosis, associated pathologies, and probiotics. Microorganisms. (2025) 13:1084. doi: 10.3390/microorganisms13051084 40431257 PMC12113704

[11] Kennedy EA King KY Baldridge MT . Mouse microbiota models: comparing germ-free mice and antibiotics treatment as tools for modifying gut bacteria. Front Physiol. (2018) 9:1534. doi: 10.3389/fphys.2018.01534. PMID: 30429801 PMC6220354

[12] Ofori-Kwafo A Sigdel I Al Mamun E Zubcevic J Tang Y . Gut-on-a-chip platforms: bridging *in vitro* and *in vivo* models for advanced gastrointestinal research. Physiol Rep. (2025) 13:e70356. doi: 10.14814/phy2.70356 40323242 PMC12051376

[13] Jennings SAV Clavel T . Synthetic communities of gut microbes for basic research and translational approaches in animal health and nutrition. Annu Rev Anim Biosci. (2024) 12:283–300. doi: 10.1146/annurev-animal-021022-025552. PMID: 37963399

[14] Delgado-Baquerizo M Singh BK Liu Y-R Sáez-Sandino T Coleine C Muñoz-Rojas M . Integrating ecological and evolutionary frameworks for SynCom success. New Phytol. (2025) 246:1922–33. doi: 10.22541/au.172797329.90740890/v1. PMID: 40177999

[15] Gallardo-Navarro O Aguilar-Salinas B Rocha J Olmedo-Álvarez G . Higher-order interactions and emergent properties of microbial communities: the power of synthetic ecology. Heliyon. (2024) 10:e33896. doi: 10.1016/j.heliyon.2024.e33896. PMID: 39130413 PMC11315108

[16] Gadhave DG Sugandhi VV Jha SK Nangare SN Gupta G Singh SK . Neurodegenerative disorders: mechanisms of degeneration and therapeutic approaches with their clinical relevance. Ageing Res Rev. (2024) 99:102357. doi: 10.1016/j.arr.2024.102357. PMID: 38830548

[17] Dash UC Bhol NK Swain SK Samal RR Nayak PK Raina V . Oxidative stress and inflammation in the pathogenesis of neurological disorders: mechanisms and implications. Acta Pharm Sin B. (2025) 15:15–34. doi: 10.1016/j.apsb.2024.10.004. PMID: 40041912 PMC11873663

[18] Xu J Lu Y . The microbiota-gut-brain axis and central nervous system diseases: from mechanisms of pathogenesis to therapeutic strategies. Front Microbiol. (2025) 16:1583562. doi: 10.3389/fmicb.2025.1583562. PMID: 40584038 PMC12202378

[19] García-Domínguez M . Neuroinflammation: mechanisms, dual roles, and therapeutic strategies in neurological disorders. Curr Issues Mol Biol. (2025) 47(6):417. doi: 10.3390/cimb47060417 40699816 PMC12191620

[20] Shi F-D Yong VW . Neuroinflammation across neurological diseases. Science. (2025) 388:eadx0043. doi: 10.1126/science.adx0043. PMID: 40536983

[21] Shimizu T Prinz M . Microglia across evolution: from conserved origins to functional divergence. Cell Mol Immunol. (2025) 22:1533–48. doi: 10.1038/s41423-025-01368-6. PMID: 41272275 PMC12660708

[22] Colonna M Butovsky O . Microglia function in the central nervous system during health and neurodegeneration. Annu Rev Immunol. (2017) 35:441–68. doi: 10.1146/annurev-immunol-051116-052358. PMID: 28226226 PMC8167938

[23] Norden DM Muccigrosso MM Godbout JP . Microglial priming and enhanced reactivity to secondary insult in aging, and traumatic CNS injury, and neurodegenerative disease. Neuropharmacology. (2015) 96:29–41. doi: 10.1016/j.neuropharm.2014.10.028. PMID: 25445485 PMC4430467

[24] Hammed O Afolabi O Ajike R Hezekiah O Alabi B Ajao D . Intestinal ischemia-reperfusion and blood-brain barrier compromise: pathways to cognitive dysfunction. Front Neurosci. (2025) 19:1597170. doi: 10.3389/fnins.2025.1597170. PMID: 40735293 PMC12303957

[25] Suzuki T . Regulation of intestinal epithelial permeability by tight junctions. Cell Mol Life Sci. (2013) 70:631–59. doi: 10.1007/s00018-012-1070-x. PMID: 22782113 PMC11113843

[26] Alahmari A . Blood-brain barrier overview: structural and functional correlation. Neural Plast. (2021) 2021:6564585. doi: 10.1155/2021/6564585. PMID: 34912450 PMC8668349

[27] Wu D Chen Q Chen X Han F Chen Z Wang Y . The blood–brain barrier: structure, regulation and drug delivery. Signal Transduction Targeted Ther. (2023) 8:217. doi: 10.1038/s41392-023-01481-w. PMID: 37231000 PMC10212980

[28] Jyoti Dey P . Mechanisms and implications of the gut microbial modulation of intestinal metabolic processes. NPJ Metab Health Dis. (2025) 3:24. doi: 10.1038/s44324-025-00066-1. PMID: 40604123 PMC12441142

[29] Nireeksha Maniangat Luke A Kumari NS Hegde MN Hegde NN . Metabolic interplay of SCFA's in the gut and oral microbiome: a link to health and disease. Front Oral Health. (2025) 6:1646382. doi: 10.3389/froh.2025.1646382. PMID: 40927228 PMC12414996

[30] Xu Z Wang T Wang Y Li Y Sun Y Qiu H-J . Short-chain fatty acids: key antiviral mediators of gut microbiota. Front Immunol. (2025) 16:1614879. doi: 10.3389/fimmu.2025.1614879. PMID: 40787446 PMC12331605

[31] Fock E Parnova R . Mechanisms of blood–brain barrier protection by microbiota-derived short-chain fatty acids. Cells. (2023) 12:657. doi: 10.3390/cells12040657. PMID: 36831324 PMC9954192

[32] Lucas LN Jillella M Cattaneo LE Gangwar B Zhang Q Clay AP . Investigation of bile salt hydrolase activity in human gut bacteria reveals production of conjugated secondary bile acids. Nat Commun. (2026) 17:3077. doi: 10.1101/2025.01.16.633392. PMID: 41730843 PMC13039732

[33] Collins SL Stine JG Bisanz JE Okafor CD Patterson AD . Bile acids and the gut microbiota: metabolic interactions and impacts on disease. (2023) 21:236–47. doi: 10.1038/s41579-022-00805-x PMC12536349 36253479

[34] Hou Y Li J Ying S . Tryptophan metabolism and gut microbiota: a novel regulatory axis integrating the microbiome, immunity, and cancer. Metabolites. (2023) 13(11):1166. doi: 10.3390/metabo13111166. PMID: 37999261 PMC10673612

[35] Bengtsson-Palme J . Microbial model communities: to understand complexity, harness the power of simplicity. Comput Struct Biotechnol J. (2020) 18:3987–4001. doi: 10.1016/j.csbj.2020.11.043. PMID: 33363696 PMC7744646

[36] Kong W Meldgin DR Collins JJ Lu T . Designing microbial consortia with defined social interactions. Nat Chem Biol. (2018) 14:821–9. doi: 10.1038/s41589-018-0091-7. PMID: 29942078

[37] Wang Z Wang S He Q Yang X Zhao B Zhang H . Ecological design of high-performance synthetic microbial communities: from theoretical foundations to functional optimization. ISME Commun. (2025) 5:ycaf133. doi: 10.1093/ismeco/ycaf133. PMID: 40860568 PMC12373479

[38] Yuan J Zhao K Tan X Xue R Zeng Y Ratti C . Perspective on the development of synthetic microbial community (SynCom) biosensors. Trends Biotechnol. (2023) 41:1227–36. doi: 10.1016/j.tibtech.2023.04.007. PMID: 37183053

[39] Shu X Liu Y . Collaboration promotes stability: insights from SynComs study. Trends Microbiol. (2025) 33:1046–7. doi: 10.1016/j.tim.2025.08.003. PMID: 40846579

[40] Williams EG Auwerx J . The convergence of systems and reductionist approaches in complex trait analysis. Cell. (2015) 162:23–32. doi: 10.1016/j.cell.2015.06.024. PMID: 26140590 PMC4493761

[41] Zhang Y Jing M Lyu L Nie L Xu X Sun R . Principles for rigorous design and application of synthetic microbial communities. Adv Sci. (2025) 13(10):e14750. doi: 10.1002/advs.202514750 PMC12915082 41420838

[42] San León D Nogales J . Toward merging bottom–up and top–down model-based designing of synthetic microbial communities. Curr Opin Microbiol. (2022) 69:102169. doi: 10.1016/j.mib.2022.102169. PMID: 35763963

[43] Yang C Sesterhenn F Bonet J van Aalen E Scheller L Abriata L . A bottom-up approach for the de novo design of functional proteins. bioRxiv. (2020). doi: 10.1101/2020.03.11.988071 33398169

[44] Gilmore SP Lankiewicz TS Wilken SE Brown JL Sexton JA Henske JK . Top-down enrichment guides in formation of synthetic microbial consortia for biomass degradation. ACS Synth Biol. (2019) 8:2174–85. doi: 10.1021/acssynbio.9b00271. PMID: 31461261

[45] Rodríguez Amor D Dal Bello M . Bottom-up approaches to synthetic cooperation in microbial communities. (2019) 9(1):22. doi: 10.3390/life9010022 PMC6462982 30813538

[46] Jiao S Yang Y Xu Y Zhang J Lu Y . Balance between community assembly processes mediates species coexistence in agricultural soil microbiomes across eastern China. ISME J. (2020) 14:202–16. doi: 10.1038/s41396-019-0522-9. PMID: 31611655 PMC6908645

[47] Smith SK de los Reyes FL . Quantifying patterns of microbial community assembly processes in bioreactors using different approaches leads to variable results. Water Res. (2025) 272:122903. doi: 10.1016/j.watres.2024.122903. PMID: 39647314

[48] Xu X Dinesen C Pioppi A Kovács ÁT Lozano-Andrade CN . Composing a microbial symphony: synthetic communities for promoting plant growth. Trends Microbiol. (2025) 33:738–51. doi: 10.1016/j.tim.2025.01.006. PMID: 39966007

[49] Zhang Y Jing M Lyu L Nie L Xu X Sun R . Principles for rigorous design and application of synthetic microbial communities. Adv Sci. (2026) 13:e14750. doi: 10.1002/advs.202514750. PMID: 41420838 PMC12915082

[50] Tarzi C Zampieri G Sullivan N Angione C . Emerging methods for genome-scale metabolic modeling of microbial communities. Trends Endocrinol Metab. (2024) 35:533–48. doi: 10.1016/j.tem.2024.02.018. PMID: 38575441

[51] Li F Chen Y Gustafsson J Wang H Wang Y Zhang C . Genome-scale metabolic models applied for human health and biopharmaceutical engineering. Quant Biol. (2023) 11:363–75. doi: 10.1002/qub2.21. PMID: 41675534 PMC12806999

[52] Cardona C Weisenhorn P Henry C Gilbert JA . Network-based metabolic analysis and microbial community modeling. Curr Opin Microbiol. (2016) 31:124–31. doi: 10.1016/j.mib.2016.03.008. PMID: 27060776

[53] Rai K Wang Y O'Connell RW Patel AB Bashor CJ . Using machine learning to enhance and accelerate synthetic biology. Curr Opin BioMed Eng. (2024) 31:100553. doi: 10.1016/j.cobme.2024.100553. PMID: 38826717

[54] Garcés-Ruiz M Díaz-Otero BG Antonielli L Saraiva JP Karpouzas D Declerck S . Machine learning for designing low-risk microbial consortia pesticides. Trends Biotechnol. doi: 10.1016/j.tibtech.2025.12.027. PMID: 41850929

[55] Piazza I Kochanowski K Cappelletti V Fuhrer T Noor E Sauer U . A map of protein-metabolite interactions reveals principles of chemical communication. Cell. (2018) 172:358–372.e323. doi: 10.1016/j.cell.2017.12.006. PMID: 29307493

[56] Kennedy SR Prost S Overcast I Rominger AJ Gillespie RG Krehenwinkel H . High-throughput sequencing for community analysis: the promise of DNA barcoding to uncover diversity, relatedness, abundances and interactions in spider communities. Dev Genes Evol. (2020) 230:185–201. doi: 10.1007/s00427-020-00652-x. PMID: 32040713 PMC7127999

[57] Sokol L Cuypers A Truong A-C Bouché A Brepoels K Souffreau J . Prioritization and functional validation of target genes from single-cell transcriptomics studies. Commun Biol. (2023) 6:648. doi: 10.1038/s42003-023-05006-7. PMID: 37330599 PMC10276815

[58] Ivanov I . Gut-host crosstalk: methodological and computational challenges. Dig Dis Sci. (2020) 65:686–94. doi: 10.1007/s10620-020-06105-9. PMID: 32016821 PMC7176510

[59] Johnson JS Spakowicz DJ Hong B-Y Petersen LM Demkowicz P Chen L . Evaluation of 16S rRNA gene sequencing for species and strain-level microbiome analysis. Nat Commun. (2019) 10:5029. doi: 10.1038/s41467-019-13036-1. PMID: 31695033 PMC6834636

[60] Pérez-Cobas AE Gomez-Valero L Buchrieser C . Metagenomic approaches in microbial ecology: an update on whole-genome and marker gene sequencing analyses. Microb Genom. (2020) 6. doi: 10.1099/mgen.0.000409. PMID: 32706331 PMC7641418

[61] Anguita-Maeso M Olivares-García C Haro C Imperial J Navas-Cortés JA Landa BB . Culture-dependent and culture-independent characterization of the olive xylem microbiota: effect of sap extraction methods. Front Plant Sci. (2020) 10:1708. doi: 10.3389/fpls.2019.01708. PMID: 32038682 PMC6988092

[62] Han D Gao P Li R Tan P Xie J Zhang R . Multicenter assessment of microbial community profiling using 16S rRNA gene sequencing and shotgun metagenomic sequencing. J Adv Res. (2020) 26:111–21. doi: 10.1016/j.jare.2020.07.010. PMID: 33133687 PMC7584675

[63] Lewis WH Tahon G Geesink P Sousa DZ Ettema TJG . Innovations to culturing the uncultured microbial majority. Nat Rev Microbiol. (2021) 19:225–40. doi: 10.1038/s41579-020-00458-8. PMID: 33093661

[64] Stewart EJ . Growing unculturable bacteria. J Bacteriol. (2012) 194:4151–60. doi: 10.1128/jb.00345-12. PMID: 22661685 PMC3416243

[65] Shayanthan A Ordoñez PAC Oresnik IJ . The role of synthetic microbial communities (SynCom) in sustainable agriculture. Front Agron. (2022) 4:896307. doi: 10.3389/fagro.2022.896307

[66] Li Y Li R Liu R Shi J Qiu X Lei J . A simplified SynCom based on core–helper strain interactions enhances symbiotic nitrogen fixation in soybean. J Integr Plant Biol. (2025) 67:1582–98. doi: 10.1111/jipb.13881. PMID: 40052412

[67] Northen TR Kleiner M Torres M Kovács ÁT Nicolaisen MH Krzyżanowska DM . Community standards and future opportunities for synthetic communities in plant–microbiota research. Nat Microbiol. (2024) 9:2774–84. doi: 10.1038/s41564-024-01833-4. PMID: 39478084

[68] Rinella MJ Reinhart KO . Mixing soil samples across experimental units ignores uncertainty and generates incorrect estimates of soil biota effects on plants. New Phytol. (2017) 216:15–7. doi: 10.1111/nph.14432. PMID: 28370145

[69] Tran TMA Gagnon VS Schelly C . A review of traditional ecological knowledge in resilient livelihoods and forest ecosystems: lessons for restoration sciences and practices. For Trees Livelihoods. (2025) 34:27–53. doi: 10.1080/14728028.2024.2408725. PMID: 37339054

[70] Sharma A Bora P . Engineering synthetic microbial communities to restructure the phytobiome for plant health and productivity. World J Microbiol Biotechnol. (2025) 41:228. doi: 10.1007/s11274-025-04460-1. PMID: 40560276

[71] Gonçalves OS Creevey CJ Santana MF . Designing a synthetic microbial community through genome metabolic modeling to enhance plant–microbe interaction. Environ Microbiome. (2023) 18:81. doi: 10.1186/s40793-023-00536-3 37974247 PMC10655421

[72] Yetgin A . Revolutionizing multi-omics analysis with artificial intelligence and data processing. Quant Biol. (2025) 13:e70002. doi: 10.1002/qub2.70002. PMID: 41675959 PMC12806145

[73] Dreyer M Berend J Labarta T Vielhaben J Wiegand T Lapuschkin S . Mechanistic understanding and validation of large AI models with SemanticLens. Nat Mach Intell. (2025) 7:1572–85. doi: 10.1038/s42256-025-01084-w. PMID: 37880705

[74] Tariq A Guo S Farhat F Shen X . Engineering synthetic microbial communities: diversity and applications in soil for plant resilience. In: Agronomy, vol. 15. (2025). p. 513.

[75] Rani J Gulia V Sangwan A Dhull SS Mandzhieva S . Synergies of traditional ecological knowledge in biodiversity conservation: a paradigm for sustainable food security. In: Jatav HS Raiput VD Minkina T , editors.Ecologically Mediated Development: Promoting Biodiversity Conservation and Food Security. Springer Nature Singapore, Singapore (2025). p. 27–49.

[76] Li C Han Y Zou X Zhang X Ran Q Dong C . A systematic discussion and comparison of the construction methods of synthetic microbial community. Synth Syst Biotechnol. (2024) 9:775–83. doi: 10.1016/j.synbio.2024.06.006. PMID: 39021362 PMC11253132

[77] Argentel-Martínez L Peñuelas-Rubio O Herrera-Sepúlveda A González-Aguilera J Sudheer S Salim LM . Biotechnological advances in plant growth-promoting rhizobacteria for sustainable agriculture. World J Microbiol Biotechnol. (2024) 41:21. doi: 10.1007/s11274-024-04231-4. PMID: 39738995

[78] Mkilima T . Synthetic biology approaches for restoring gut microbial balance and engineering disease-specific microbiome therapeutics. Microb Pathogen. (2025) 207:107931. doi: 10.1016/j.micpath.2025.107931. PMID: 40716471

[79] da Silva EC Guerrero-Moreno MA Oliveira FA Juen L de Carvalho FG Barbosa Oliveira-Junior JM . The importance of traditional communities in biodiversity conservation. Biodivers Conserv. (2025) 34:685–714. doi: 10.1007/s10531-024-02999-3. PMID: 30311153

[80] van Leeuwen PT Brul S Zhang J Wortel MT . Synthetic microbial communities (SynComs) of the human gut: design, assembly, and applications. FEMS Microbiol Rev. (2023) 47:fuad012. doi: 10.1093/femsre/fuad012. PMID: 36931888 PMC10062696

[81] Wu W-L Adame MD Liou C-W Barlow JT Lai T-T Sharon G . Microbiota regulate social behaviour via stress response neurons in the brain. Nature. (2021) 595:409–14. doi: 10.1038/s41586-021-03669-y. PMID: 34194038 PMC8346519

[82] Jiang X Peng Z Zhang J . Starting with screening strains to construct synthetic microbial communities (SynComs) for traditional food fermentation. Food Res Int. (2024) 190:114557. doi: 10.1016/j.foodres.2024.114557. PMID: 38945561

[83] Gonzalez-Garcia J Delgado-Villalobos A Hernandez-Ruiz G Díaz-Torres O García-Cayuela T Gradilla-Hernández MS . Synthetic microbial communities as novel models to study gut microbiome–host interactions in metabolic diseases. Discover Endocrinol Metab. (2025) 1:12. doi: 10.1007/s44417-025-00012-1. PMID: 30311153

[84] Li CV Knoblich JA . Advancing autism research: insights from brain organoid modeling. Curr Opin Neurobiol. (2025) 92:103030. doi: 10.1016/j.conb.2025.103030. PMID: 40279814

[85] Kumar S Ramos E Hidalgo A Rodarte D Sharma B Torres MM . Integrated multi-omics analyses of synaptosomes revealed synapse-associated novel targets in Alzheimer’s disease. Mol Psychiatry. (2025) 30:5121–36. doi: 10.1038/s41380-025-03095-w. PMID: 40581657 PMC12532717

[86] Kaul M Mukherjee D Weiner HL Cox LM . Gut microbiota immune cross-talk in amyotrophic lateral sclerosis. Neurotherapeutics. (2024) 21:e00469. doi: 10.1016/j.neurot.2024.e00469. PMID: 39510899 PMC11585889

[87] Banerjee S Schlaeppi K van der Heijden MGA . Keystone taxa as drivers of microbiome structure and functioning. Nat Rev Microbiol. (2018) 16:567–76. doi: 10.1038/s41579-018-0024-1. PMID: 29789680

[88] Vlachas PR Arampatzis G Uhler C Koumoutsakos P . Multiscale simulations of complex systems by learning their effective dynamics. Nat Mach Intell. (2022) 4:359–66. doi: 10.1038/s42256-022-00464-w. PMID: 37880705

[89] Tan SH Karri V Tay NWR Chang KH Ah HY Ng PQ . Emerging pathways to neurodegeneration: dissecting the critical molecular mechanisms in Alzheimer’s disease, Parkinson’s disease. Biomedicine Pharmacotherapy. (2019) 111:765–77. doi: 10.1016/j.biopha.2018.12.101. PMID: 30612001

[90] Trisal A Singh I Garg G Jorwal K Singh AK . Gut–brain axis and brain health: modulating neuroinflammation, cognitive decline, and neurodegeneration. 3 Biotech. (2024) 15:25. doi: 10.1007/s13205-024-04187-0. PMID: 39735610 PMC11680542

[91] Chen L Zhernakova DV Kurilshikov A Andreu-Sánchez S Wang D Augustijn HE . Influence of the microbiome, diet and genetics on inter-individual variation in the human plasma metabolome. Nat Med. (2022) 28:2333–43. doi: 10.1038/s41591-022-02014-8. PMID: 36216932 PMC9671809

